# Heavy-Metal-Free Epoxy Vitrimers Enhanced with Liquid Crystalline Phase †

**DOI:** 10.3390/ma19163471

**Published:** 2026-08-17

**Authors:** Maciej Kisiel

**Affiliations:** Department of Industrial and Materials Chemistry, Faculty of Chemistry, Rzeszów University of Technology, al. Powstańców Warszawy 6, 35-029 Rzeszów, Poland; m.kisiel@prz.edu.pl; Tel.: +48-177432572

**Keywords:** epoxy resin, self-healing, DSC, POM, liquid crystals, polymer network

## Abstract

**Highlights:**

- Self-healing is facilitated by excess hydroxyl groups from glycerol, without a heavy metal catalyst.
- Self-healing is enhanced by the addition of liquid crystalline epoxy resin.
- Liquid crystalline epoxy resin can be a single self-healing promoter.
- The addition of liquid crystalline epoxy resin elasticizes the network.
- The obtained vitrimers allow the design of self-healing coatings with a prolonged lifespan.

**Abstract:**

A new, heavy-metal-catalyst-free epoxy vitrimer system modified with a liquid crystalline epoxy resin (LCER) is presented. A critical innovation in this work was the replacement of conventional toxic transesterification catalysts, such as zinc compounds, with an excess of a bio-based compound: glycerol. Two anhydrides, maleic anhydride (MA) and phthalic anhydride (PA), were evaluated as curing agents to determine their impact on the thermal and self-healing properties of the resulting networks. The study revealed that the systems cured with an aliphatic anhydride exhibited superior self-healing characteristics compared to their aromatic counterparts. Moreover, the use of MA allowed for a lower curing temperature. Differential scanning calorimetry (DSC) was employed to analyze the thermal behavior of the resin and its compatibility with the epoxy vitrimer network, as well as the curing process itself and the thermal characteristics of the obtained polymer network. Hot-stage-polarized optical microscopy (HS-POM) was used to confirm the formation of liquid crystalline phases of the LCER and to track the healing process. Furthermore, it was observed that in selected formulations, the addition of LCER itself was sufficient to induce self-healing capabilities without the necessity of adding multi-hydroxyl alcohol.

## 1. Introduction

Epoxy resins have proven to be indispensable chemicals due to their excellent properties that cannot be surpassed by any other materials in this area of application. Their importance is backed by a plurality of scientific works dedicated to this manner [1,2,3]. One of their greatest weaknesses is their inability to be reprocessed and recycled, which makes them single-use synthetic materials. Growing ecological and environmental awareness has been the cause of the attempts to retain the durable and strong thermosetting epoxy network while enabling the self-healing or even recycling and reprocessing of this network. The result of this experiment is epoxy vitrimers—thermosets with the unique ability to reprocess, recycle, and self-heal without compromising network integrity. Unlike traditional thermosetting polymers, which are typically insoluble and infusible after curing, vitrimers contain dynamic covalent bonds that can undergo topological rearrangements upon stimulation (e.g., heat), enabling material circularity and extending product lifecycle [4,5,6,7]. They can still be used as materials with excellent thermal properties and elastomeric behavior [8]. With proper synthetic route and planning they offer a wide range of possible applications due to fast stress relaxation [9,10], malleability in moderate temperatures [10], improved reprocessability [11,12,13] with toughness and rigidity controlled by water content [12], good mechanical properties [14], and even shape memory [15]. Material properties can also be tuned further by changing the ratio of the constituents [16].

The mechanisms of vitrimer healing are primarily based on their ability to reconfigure and reshuffle their covalent networks under the influence of external stimuli, such as heat and pressure. Key mechanisms include dynamic covalent bond exchange [7], like transesterification [17,18,19,20,21] and reactions based, for example, on rearrangement of disulfide [9,22], imine [23], boronic ester [24], alkoxyamine [25], carbonate [26] amine [27,28] or siloxane [15] groups. In the case of most of these systems, a catalyst is needed for efficient and effective healing of the network. Those catalytic systems usually consist of toxic, hard-to-isolate compounds, like zinc salts [29,30] and polymeric compounds containing zinc [31] or organocatalysts, like 1,5,7-triazabicyclo[4.4.0]dec-5-ene (TBD) [10], imidazoles [32] or triphenylphosphines [33]. There have been some attempts to formulate non-catalytic vitrimeric materials with the use of hyperbranched epoxy prepolymers with an excess of hydroxyl groups [34,35] or glycerol as a hydroxyl-rich transesterification promoter [36]. This latter work was the inspiration for the presented study, with the aim to further enhance the reprocessability and healing properties of the vitrimer with the addition of the LCER. Connecting concepts of the vitrimers and liquid crystals (LCs) has been explored before with different compositions, with good results, giving materials with superior reprocessability [37], high thermal conductivity [37,38,39], tensile strength and toughness, as well as shape memory effect [39] and negative Poisson’s ratio [40]. Some recyclable systems are suitable for 3D printing [41].

This work aims to explore the area of glycerol-induced healing of the vitrimers with enhancement based on the addition of synthesized LCER. Our resins have shown interesting electrical, thermal and morphological features [42,43,44], which makes them good candidates for incorporation into vitrimer networks.

## 2. Materials and Methods

### 2.1. Materials

**Figure 1:**
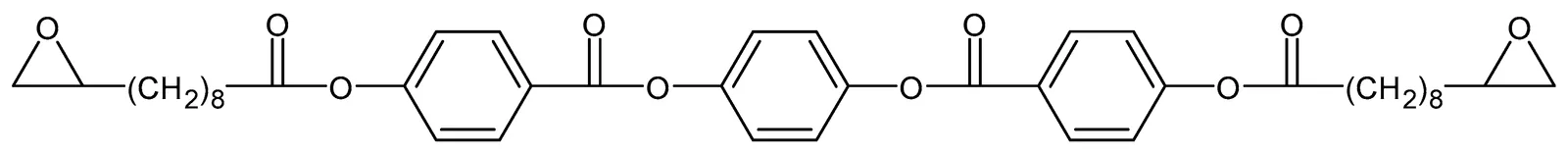
Chemical structure of MU22 LCER.

- Epidian^®^ 6 (EP6), Sarzyna Chemical, Nowa Sarzyna, Poland;
- Glycerol (Gl), 99%, Sigma Aldrich, Taufkirchen, Germany;
- Maleic anhydride (MA), 99%, Sigma Aldrich, Taufkirchen, Germany;
- Phtalic anhydride (PA), 99%, Sigma Aldrich, Taufkirchen, Germany;
- MU22 liquid crystalline epoxy resin, synthesized, with the structure presented in Figure 1.

### 2.2. Methods

#### 2.2.1. Synthesis

Synthesis of the LCER was performed using a procedure described elsewhere [45].

#### 2.2.2. Sample Preparation

**Figure 2:**
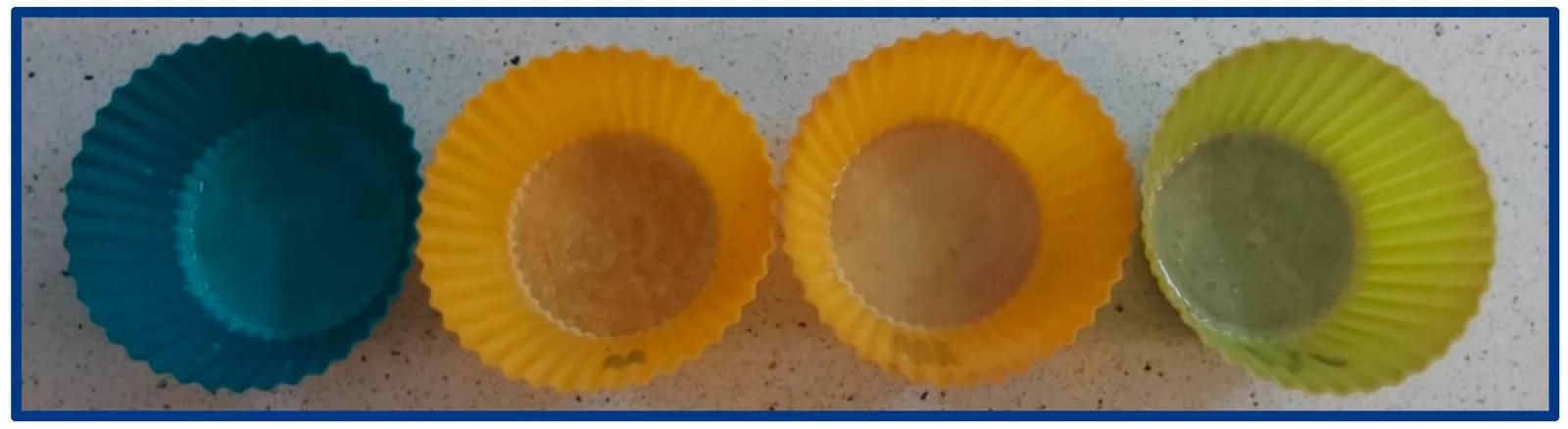
Photograph of examples of the cured samples.

Samples were prepared by mixing the reagents with the amounts presented in Table 1. The molar amount of the anhydride was equal to the total epoxy group amount (EP6 + MU22). LCER addition was 10% of the mass of the EP6. Glycerol was added as a 0.5 molar amount of the total epoxy group. The investigation of the optimal glycerol content (0.25, 0.5, 0.75 molar ratio) was also conducted. Preparation started with milling of the anhydride in a coffee grinder. Small portions of the mixtures were placed in silicon muffin cups and cured in a muffle furnace for 1 h at 150 °C for MA and at 200 °C for PA compositions. Curing started at room temperature, with a heating rate of 10 °C min^−1^. A photograph of the samples is presented in Figure 2.

#### 2.2.3. Differential Scanning Calorimetry (DSC) Analysis

DSC analysis was performed using a Mettler Toledo (Columbus, OH, USA) DSC1 apparatus with Star^e^System 17.2, in a nitrogen atmosphere with a flow of 60 mL min^−1^, with liquid nitrogen as a cooling agent. The temperature range of the analysis was set individually for each sample and composition and is visible on every thermal curve. The heating rate was 10 °C min^−1^. Samples were prepared by weighing the materials in the standard 40 µL aluminum crucibles.

#### 2.2.4. Hot-Stage-Polarized Optical Microscopy (HS-POM) Analysis

HS-POM analysis was performed using an Opta-Tech (Warsaw, Poland) LAB40 microscope coupled with a Linkam (Salfords, Great Britain) LTS420 heating stage equipped with a liquid nitrogen pump for stable cooling. Images were captured using an Opta-Tech (Warsaw, Poland) MI20 digital camera. Instruments were handled using LINK 1.15 and Capture 2.4 software. The temperature ranges of observations were 25–150 °C for MA and PA compositions and 0–200 °C for the LCER monomer. The heating rate was 10 °C min^−1^. For determination of the self-healing process, the surface of the polymer network was scratched with a scalpel and placed on the microscope slide, and the size and character of the scratch were observed. All photographs were taken at 200× magnification.

#### 2.2.5. Use of the GenAI

GenAI (NotebookLM, Mountain View, CA, USA) was used solely in preparing graphics for the graphical abstract.

## 3. Results

### 3.1. Characterization of the LCER Monomer

**Figure 3:**
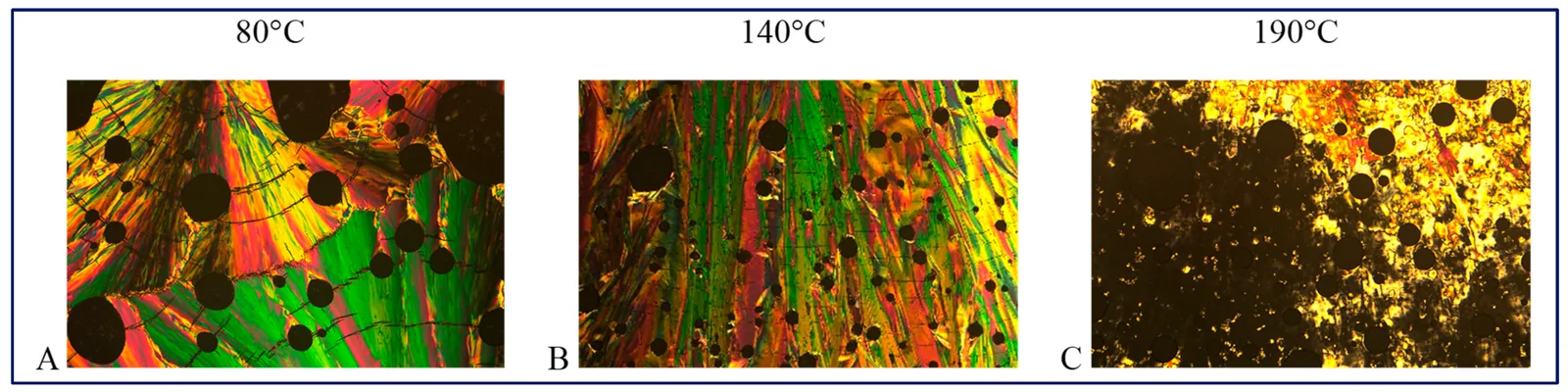
Microscopic textures of the MU22 detected during HS-POM observations.

The HS-POM microphotographs with a magnification of 200× (Figure 3) and the DSC thermal curves in the temperature range of 0–200 °C (Figure 3) were recorded to investigate thermal properties and phase character of the synthesized LCER.

**Figure 4:**
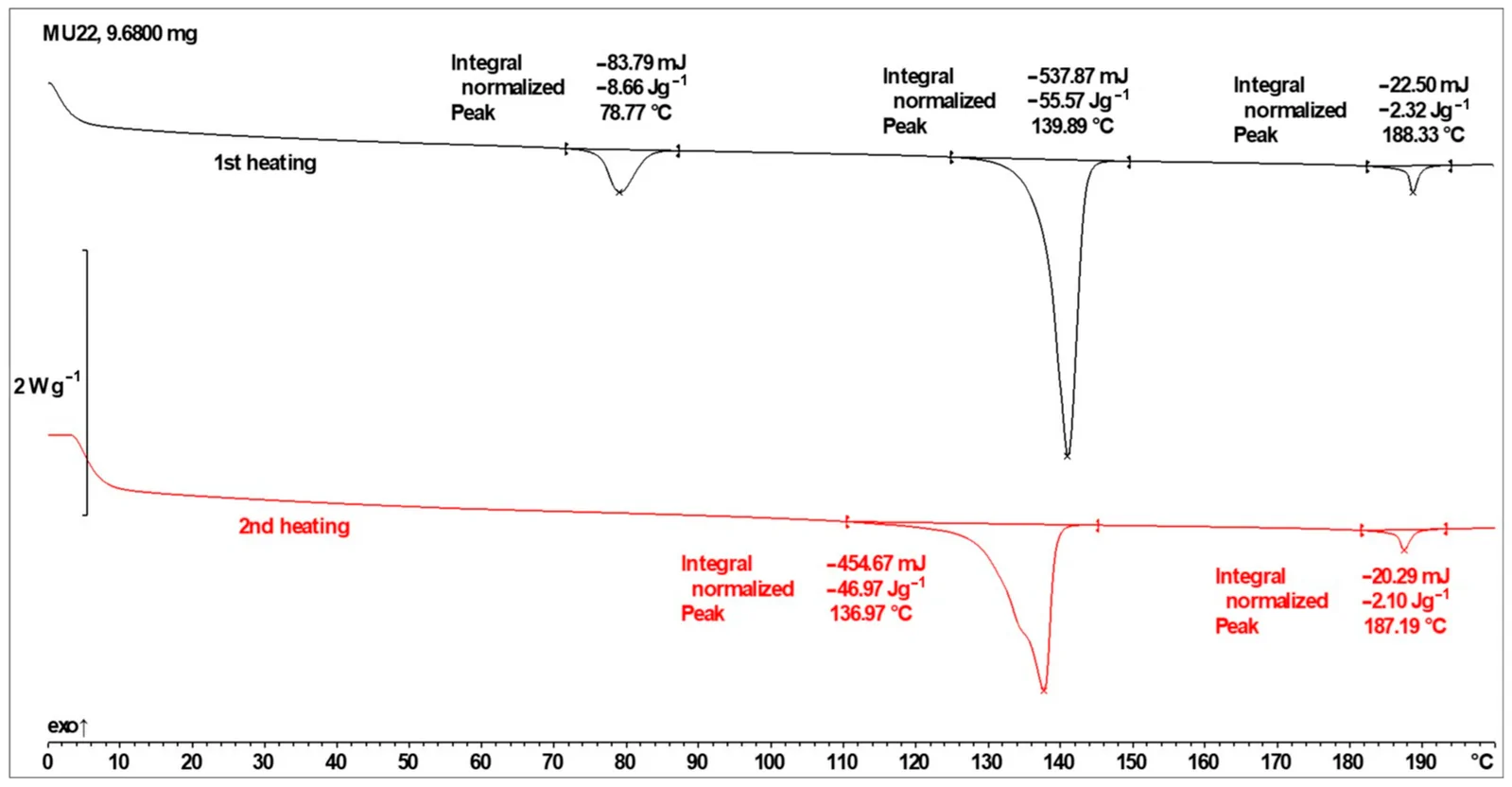
DSC thermal curve of the MU22 monomer.

Based on the data collected during the POM observations (Figure 3) and the DSC analysis (Figure 4), the thermal properties and LC nature of the compound were determined and confirmed.

### 3.2. HS-POM Observations of the Scratched Polymer Surfaces

**Figure 5:**
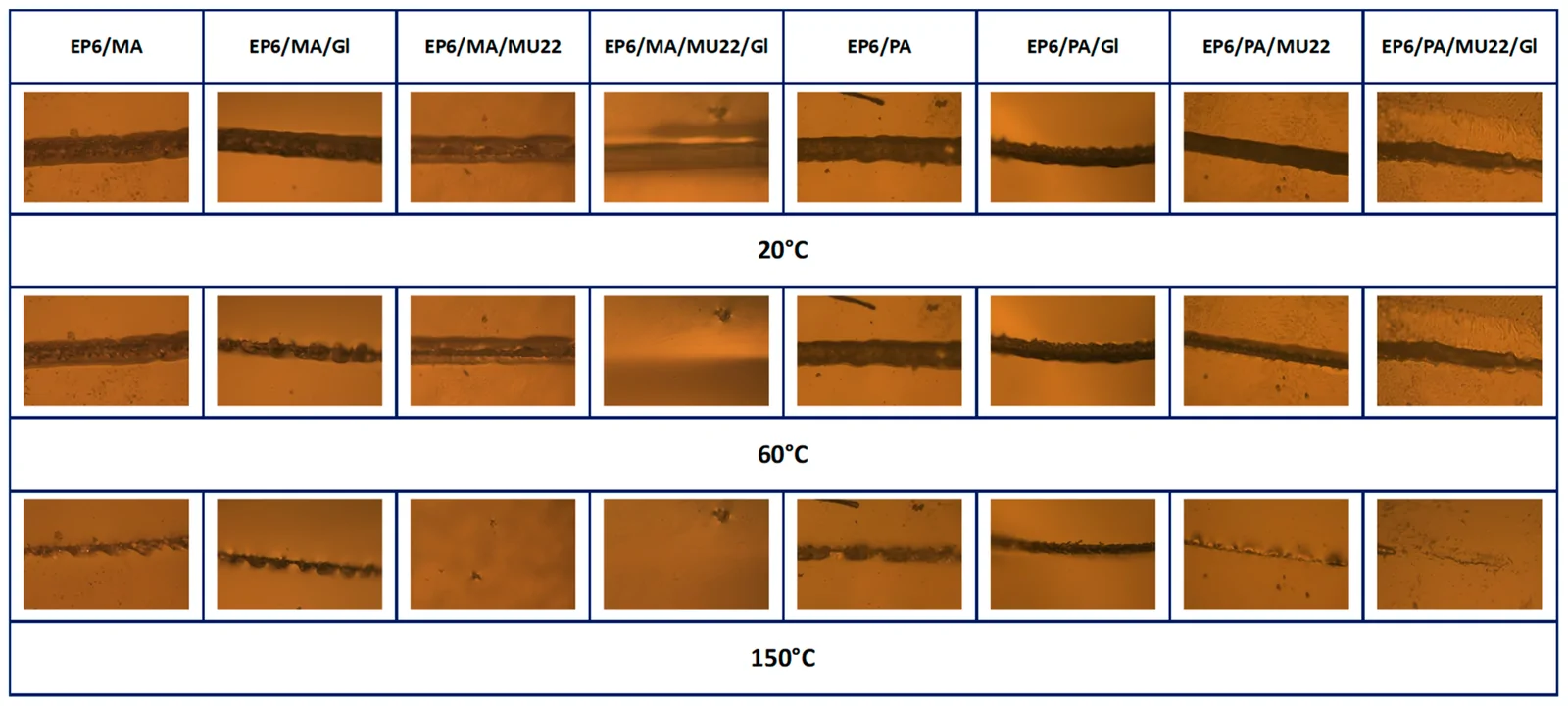
Microphotographs of the prepared samples illustrating the self-healing process.

The self-healing process was observed during POM experiments, and the results are presented in Figure 5. All photographs were taken during the heating cycles with a rate of 10 °C/min.

### 3.3. DSC Analysis of the Obtained Polymer Networks

**Figure 6:**
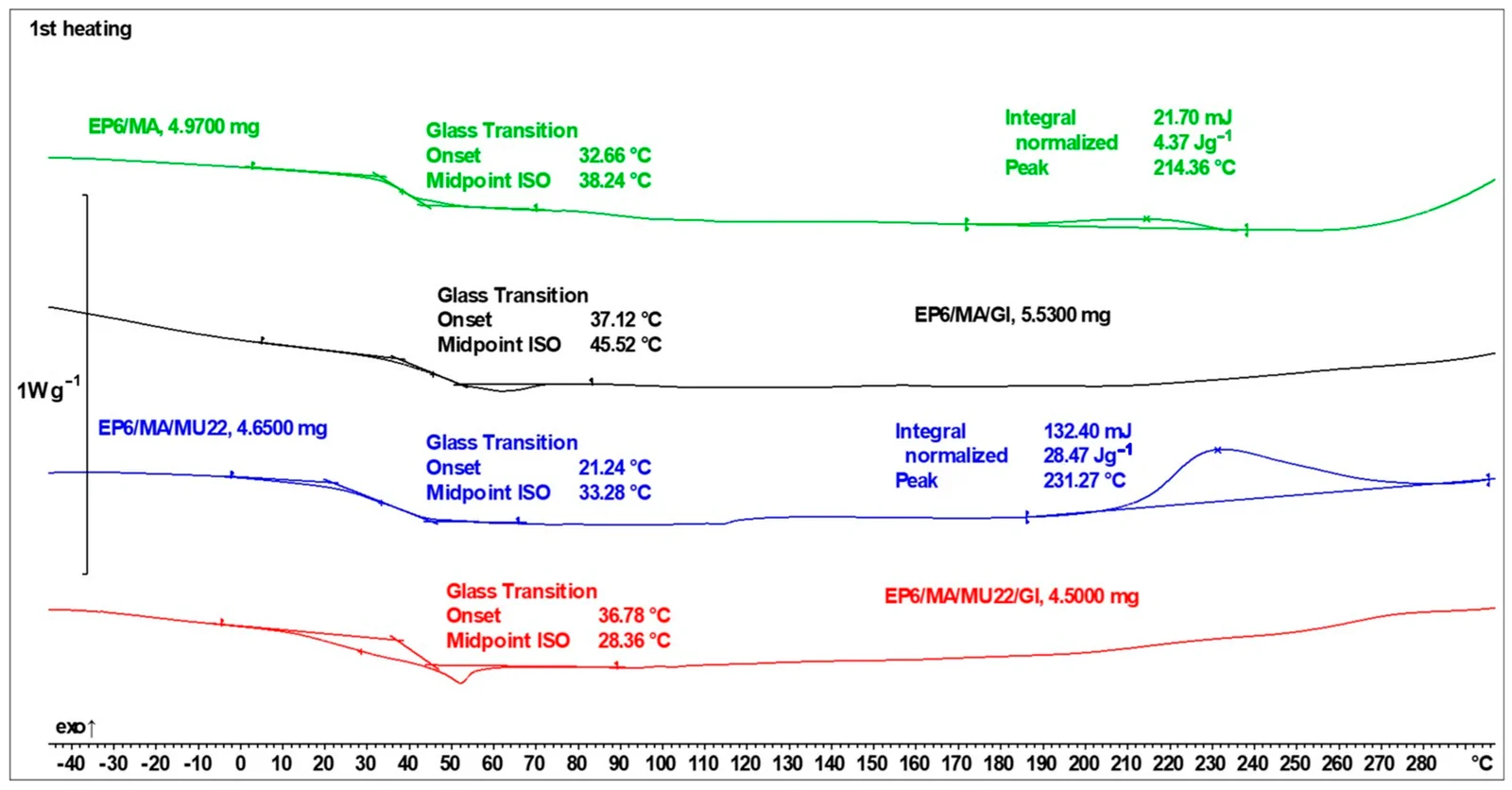
DSC thermal curves of the vitrimer networks containing MA—1st heating run.

**Figure 7:**
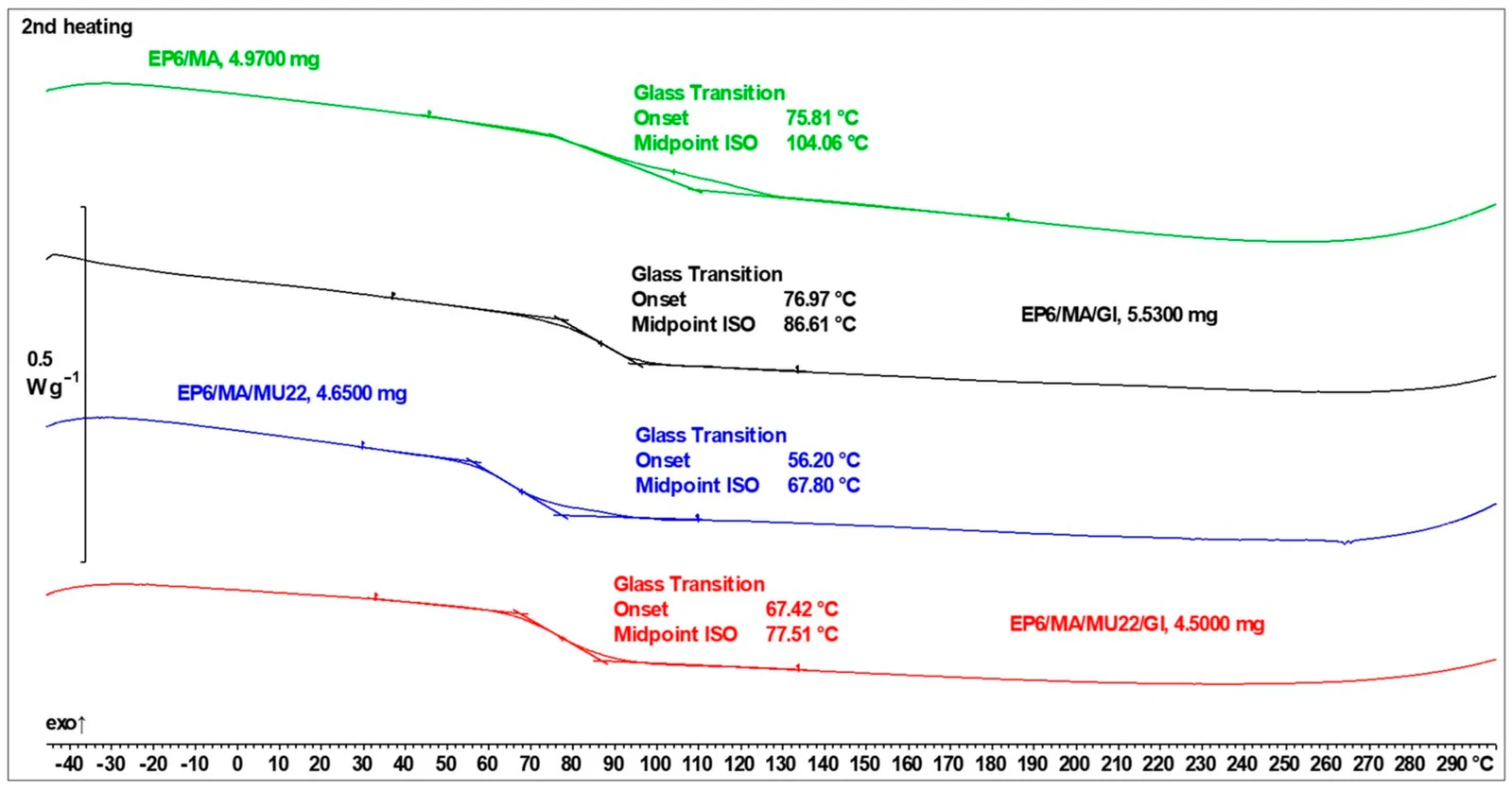
DSC thermal curves of the vitrimer networks containing MA—2nd heating run.

**Figure 8:**
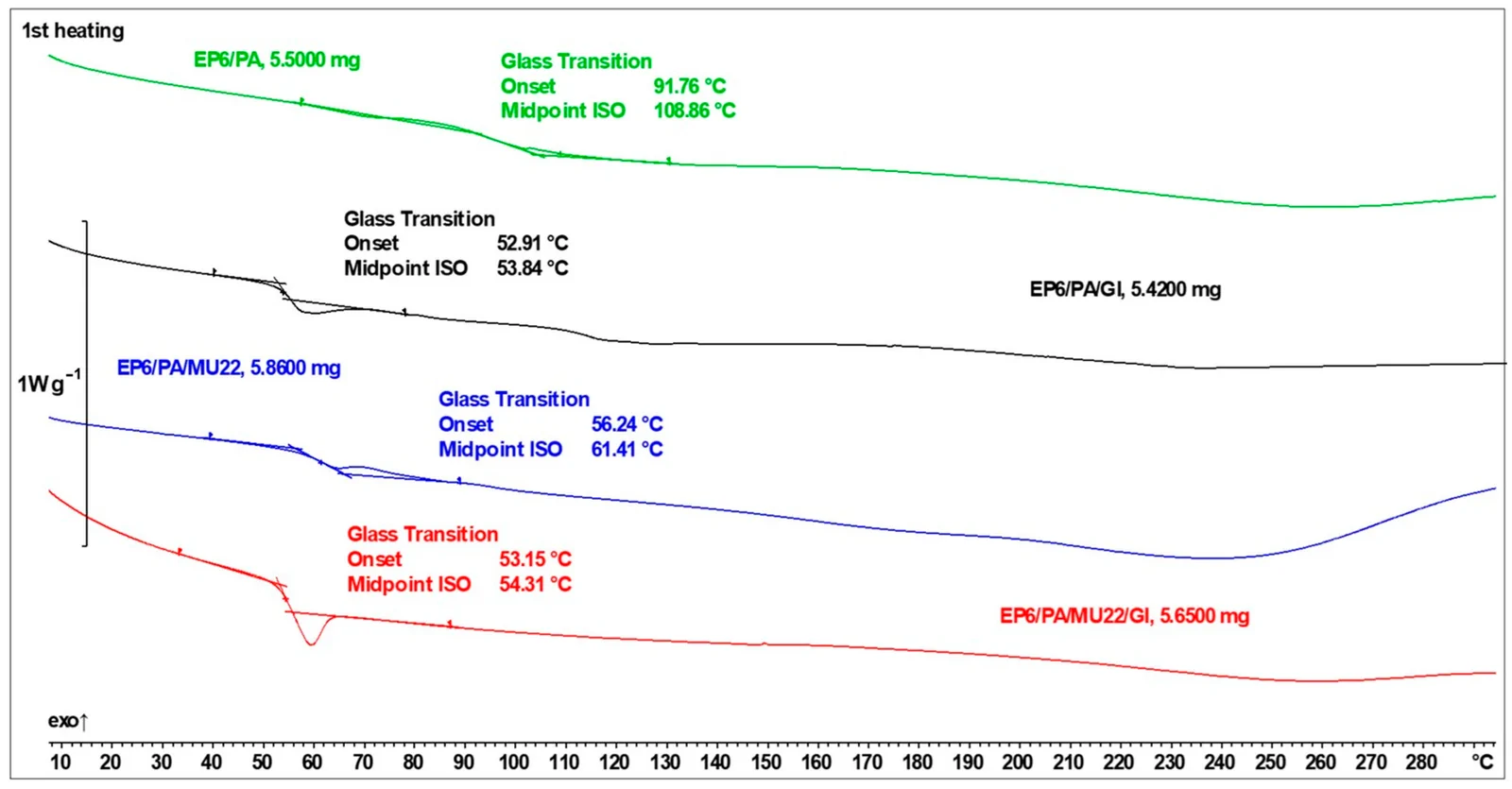
DSC thermal curves of the vitrimer networks containing PA—1st heating run.

**Figure 9:**
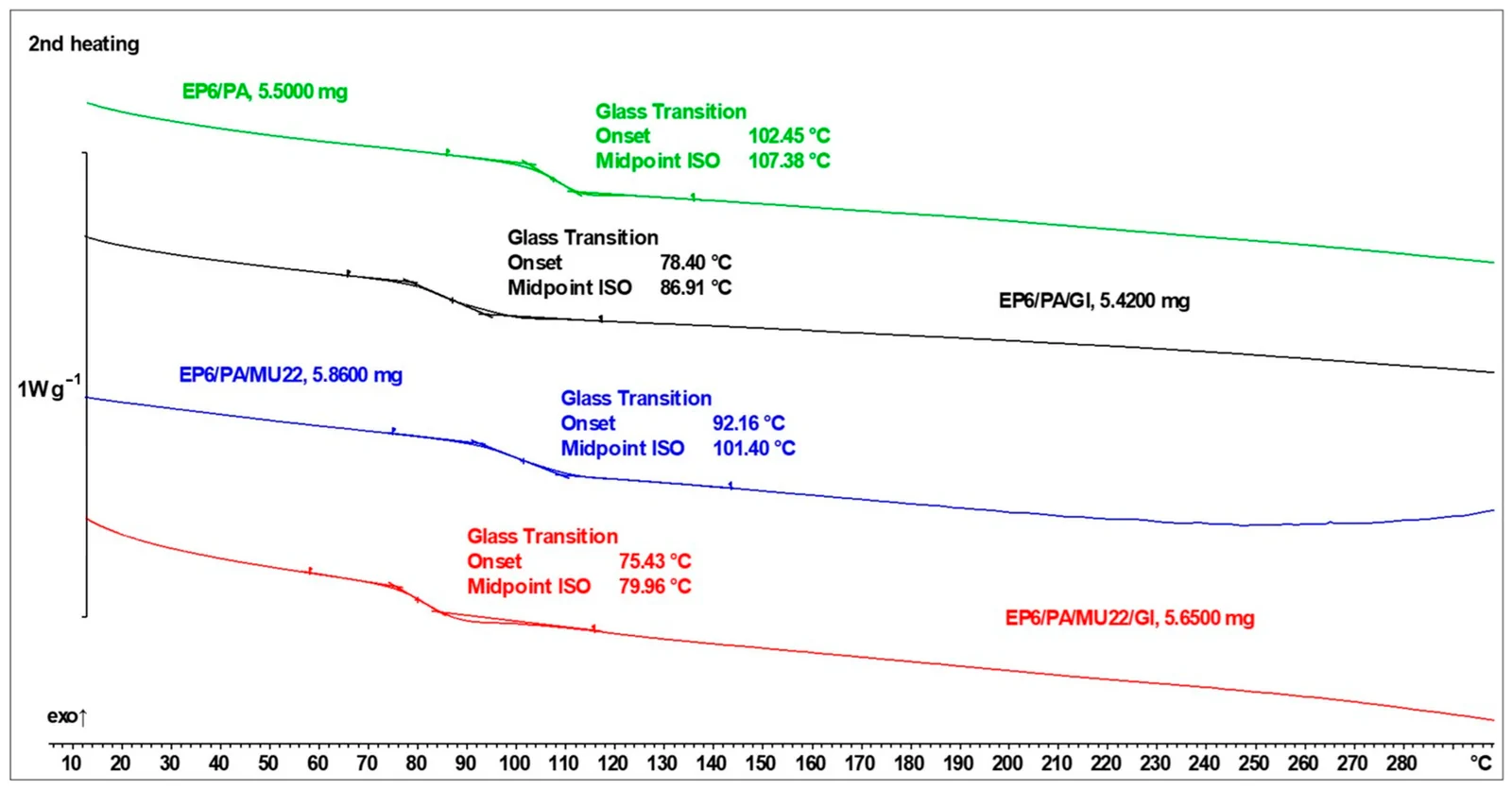
DSC thermal curves of the vitrimer networks containing PA—2nd heating run.

The analysis of the two DSC runs of the synthesized networks was conducted, and the results are presented in Figure 6, Figure 7, Figure 8 and Figure 9.

### 3.4. HS-POM and DSC Analysis of the Samples with Various Glycerol Content

**Figure 10:**
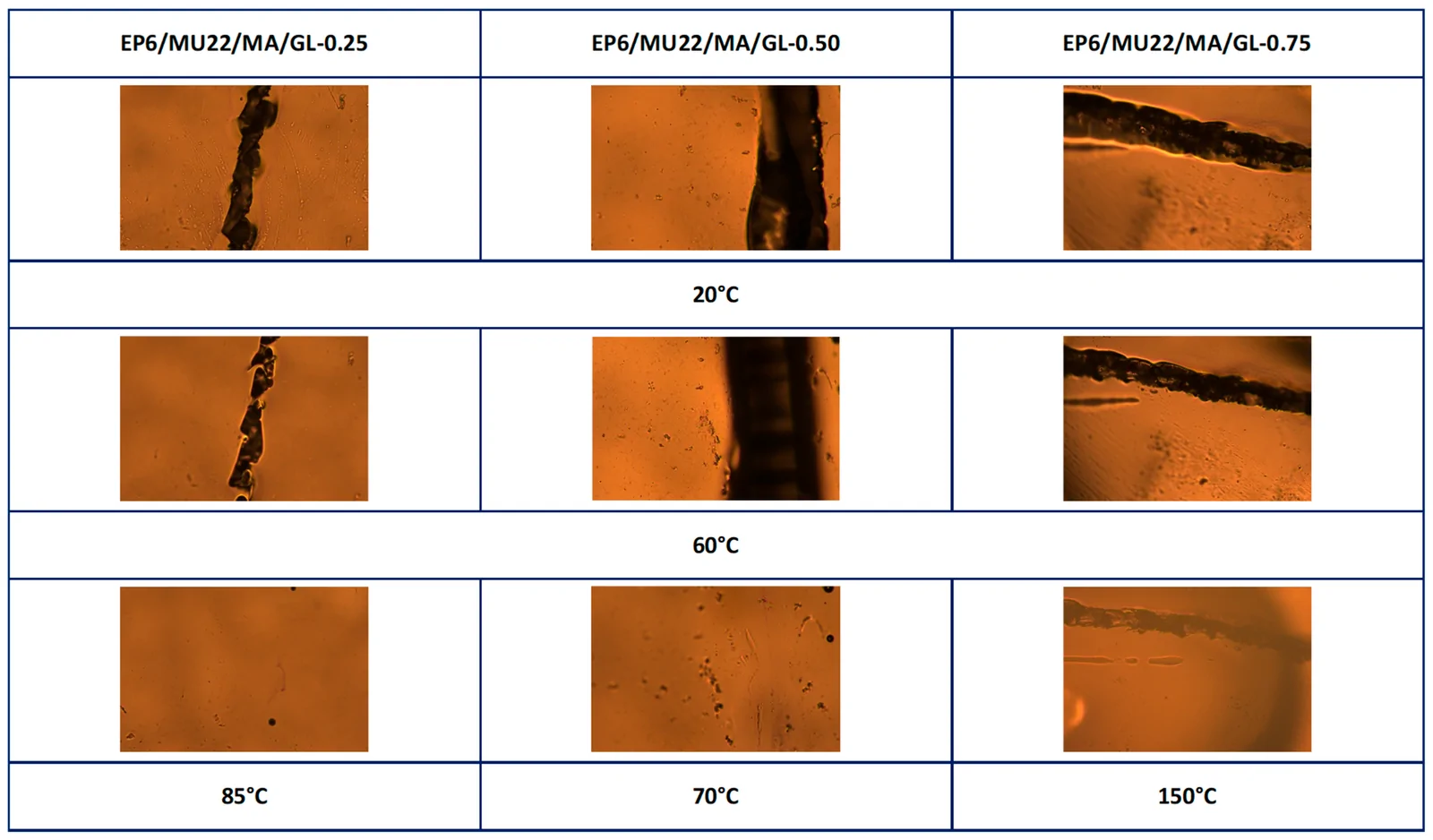
Microphotographs illustrating the effect of glycerol content on self-healing.

Another batch of mixtures was prepared to assess the effect of the amount of alcohol on the healing and thermal properties of the networks, and the experiments were conducted in the same manner. The HS-POM photographs are presented in Figure 10. The temperature in the last row of the pictures is the lowest temperature that allowed complete healing or, in the case of residual scratch, the temperature at the end of the observations (150 °C). Only the MA was used for this study, as it was confirmed that aliphatic hardener gives better results.

**Figure 11:**
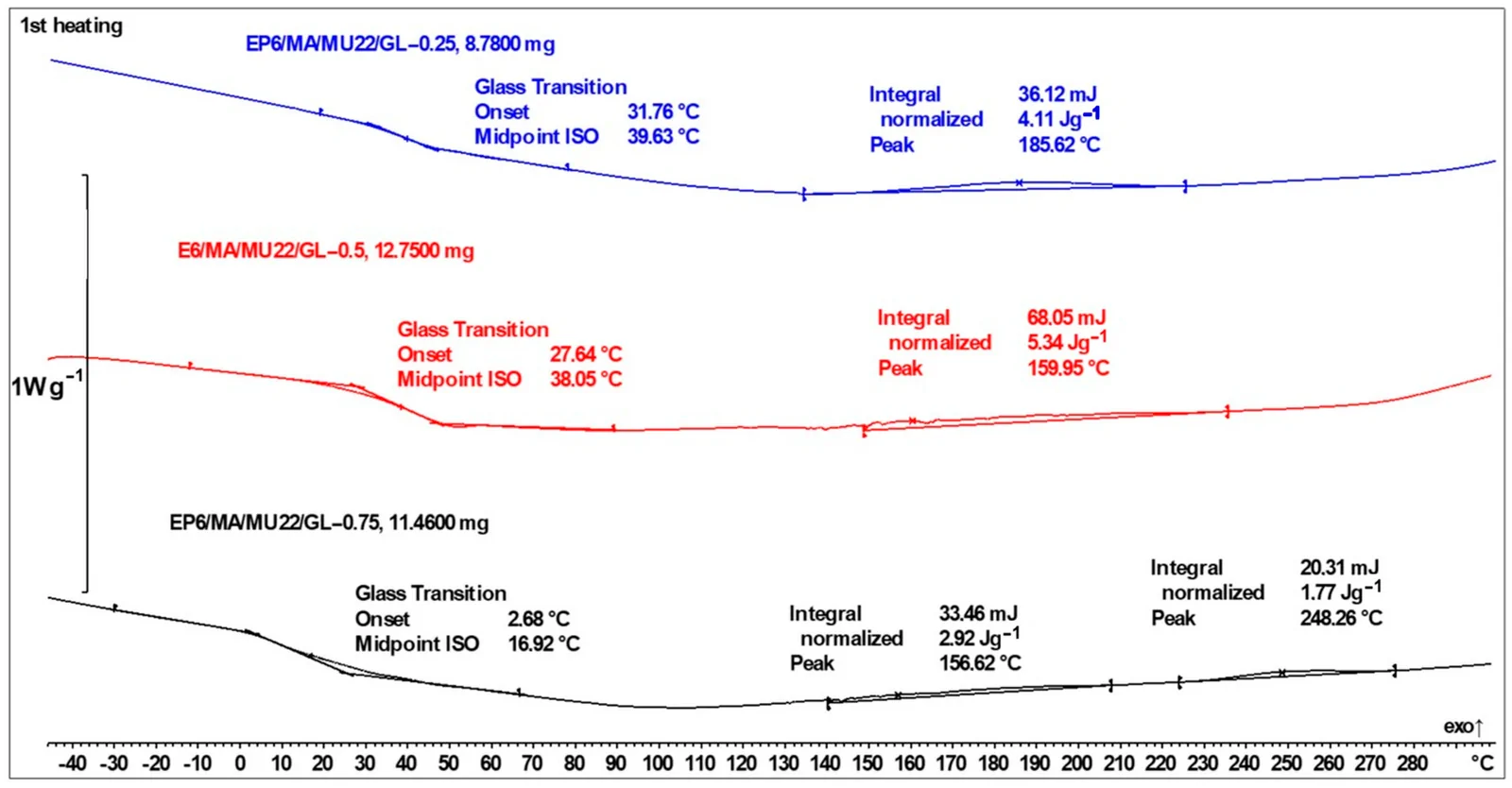
DSC thermal curves of the vitrimer networks with different glycerol content—1st heating.

**Figure 12:**
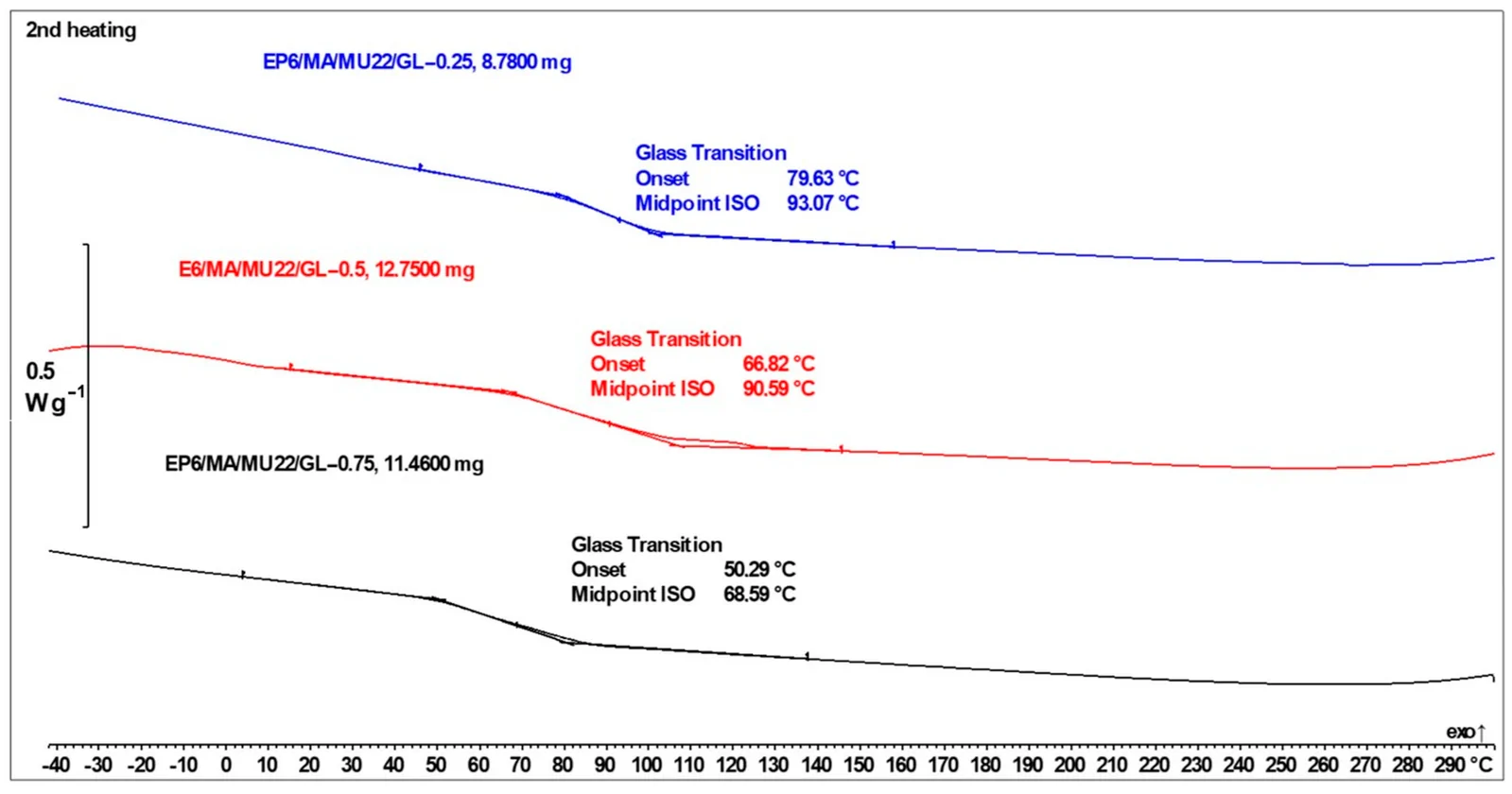
DSC thermal curves of the vitrimer networks with different glycerol content—2nd heating.

DSC studies aimed at determination of the *T_g_* and any other important thermal processes of those samples were also performed (Figure 11 and Figure 12).

## 4. Discussion

### 4.1. Thermal Characteristics of the LCER Monomer

Based on the data collected during the POM observations (Figure 3) and the DSC analysis (Figure 4), the thermal properties and LC nature of the compound were determined and confirmed. The analysis of the obtained data confirms the purity and the LC nature of the obtained resin. Both thermal characteristics and the mesophase type were compliant with experiments conducted previously [46]. The results show polymorphic transition occurring at around 80 °C, then the transition to nematic liquid crystal [47] is present at around 140 °C, and, finally, isotropization is taking place at 190 °C. In the second heating run, the polymorphic transition is not present, which suggests that the first crystalline form of the compound is not kinetically favored. However, the presence of second and third peaks on the DSC curve confirms the stability and purity of the LCER. Based on these results, we established a curing temperature window between 140 and 190 °C to facilitate working of the MU22 in the LC state.

### 4.2. Analysis of the Healing Properties of the Prepared Samples

The self-healing process was observed during POM experiments, and the results are presented in Figure 5. The POM experiments brought evidence of the self-healing properties of the investigated epoxy compositions. As a control, the EP/MA and EP/PA samples were observed. The lack of healing promoter (glycerol—as the agent delivering abundance of hydroxyl groups ready for transesterification) caused a very limited healing image. The scratch on those samples narrowed, and its edges rounded only at very high temperature; this is caused also by elastification of the material above the glass transition temperature *T_g_*. Changes are more visible for MA samples, as the aliphatic chain offers more elasticity than the aromatic PA structure.

In the case of the samples containing glycerol (EP6/MA/Gl and EP6/PA/Gl), the healing process is slightly more visible, but the results are still not satisfactory. The scratch remained present with the effect similar to control samples, but present at lower temperatures. Thus, the healing is occurring earlier, but the process is not complete. Again, the properties are slightly better for the aliphatic hardener.

Doping the mixtures with LC and its “flowing-like” character (samples EP6/MA/MU22/Gl and EP6/PA/MU22/Gl) proved to be an effective enhancement of the self-healing nature of the networks. In both cases, the healing is complete or almost complete (visible “scar” on the sample with PA at 150 °C). Fully healed EP6/MA/MU22/Gl material was unfortunately flawed in other ways. It was not completely solid and had some gel features—it was a bit sticky and difficult to handle, so it could be problematic to use it at ambient temperature. Storing this material in the fridge before preparing the sample solidified the network and made operation with it more convenient. These gel-like properties can be intuitively noticed on the microphotographs. Nonetheless, the healing process was complete, and in the case of MA anhydride, even at around 60 °C.

Two additional mixtures were prepared (samples EP6/MA/MU22 and EP6/PA/MU22) to assess the effect of the LCER alone on the self-healing properties. Those samples are arguably the most valuable finding of this research as they give very similar results to the more complicated and harder-to-manage compositions also containing glycerol. In the case of the aromatic curing agent, the residual scar is only a bit more visible. The most promising result was found for the sample EP6/MA/MU22. The polymer network was solid at ambient temperature, the sample was easy to prepare, and it allowed complete healing. The healing temperature is slightly higher, but it proved that the liquid part of the LC’s nature (possibility of flowing) is capable of transforming the traditional and cheap epoxy network into effective vitrimer only by a small addition of the liquid crystalline reactive agent.

### 4.3. Thermal Properties of the Vitrimers

Vitrimers based on the aliphatic hardener have relatively low *T_g_* in the first heating cycle (Figure 6). There is a visible plasticizing effect of the addition of the LCER due to their long, aliphatic spacers, which lower the *T_g_* in comparison to the non-LC samples. In the case of networks without the mesophase, the glycerol addition increases the *T_g_*, probably causing more network density by the incorporation of more reaction sites. In the case of networks without the alcohol, there are peaks with maxima placed above 200 °C, which can be attributed to post-curing or transesterification processes connected to the healing rearrangement. This was expected, as the mixtures are non-stoichiometric, and with the rise in the temperature and drop in the viscosity, the residual functional groups are more accessible, especially when there are flowing LC domains. All of the samples present a slight rise in the DSC curves above the *T_g_*, which shows the phenomena described in the literature [38]—the dehydration of the free hydroxyl groups. Additional evidence of this, consistent with the cited article, is the significant rise in the *T_g_* in the second heating run (Figure 7). There was also organoleptic evidence of the dehydration, shown by water condensing on the microscope slide during HS-POM experiments.

Thermal curves recorded for MA-containing compositions in the second heating (Figure 7) are a typical representation of the hardened epoxy with only the glass transition temperature easily possible to detect. The value of them rose significantly, and there are no other visible effects on the curves than the change in the slope of the curves close to 300 °C. This can be explained by further dehydration or the beginning of the thermal degradation of the material.

The DSC results of the PA-containing matrices (Figure 8 and Figure 9) are similar in interpretation to those with the MA. The *T_g_* values are higher, due to the stiff, aromatic structure of PA, so both agents with the aliphatic chains (MU22, glycerol) have a plasticizing effect. In this case, the glycerol has more profound action on this property than the LC resin. There are also signs of some post-curing, transesterification and dehydration reactions in the first heating run after the synthesis in the furnace. Those reactions are proved by a rise in the *T_g_* in the second heating, with the exception of the pure EP6/PA network, which did not change the glass transition temperature in the course of the DSC experiments, which proves complete curing in the furnace. This is somewhat expected, as this matrix should not present any healing-induced or rearranging reactions of the network. There is a noticeable difference compared to the MA compositions. Only the EP6/PA/MU22 sample presents the rise in the thermal curve in the second heating, which may be evidence of the healing capacity of the LC resin and additional proof of the stronger vitrimeric properties of the MA samples. An interesting fact can be seen in Figure 8, in the case of EP6/PA/Gl DSC curve. There is a slightly visible change at around 115 °C, which indicates a second amorphous phase with increased rigidity. This residual glass transition temperature could provide evidence that with the use of the less reactive aromatic hardener the mixture undergoes some block-like formation with dominant, more elastic character (EP6/PA/Gl) and residual, stiff segments (EP6/PA). The effect, which may be described as the second *T_g_*, has low intensity, so the formation of stiffer segments is very limited.

Furthermore, DSC curves presented, for example, in Figure 8 show the ongoing enthalpy relaxation after reaching the glass transition temperature. This phenomenon is the most profound for the samples containing glycerol. The addition of this alcohol dramatically increases the number of the hydroxyl groups in the polymer structure. Those can form hydrogen bonds, thus making the network less uniform. This annealing is a sign of the process of physical aging. Glycerol-containing formulations are expected to have some annealing processes and are sometimes even used as model mixtures in the physical aging studies [48]. The intensity of the relaxation is, however, quite limited, and this process is erased via heating, which is necessary for healing of the defect and will happen often during the lifecycle of the polymer. Considering this, it should not deteriorate the properties of the coating.

For all of the samples, despite the notion from the DSC curves that the curing reaction is complete, especially after the second heating (lack of post-curing, exothermic peak), there are always some intact epoxy groups left protected sterically by the formed network. Those unreacted functionalities can also take part in the reorganization of the network, resulting in the self-healing of the coating.

A summary of the thermal properties of the samples is presented in Table 2.

There is also a need to discuss the possibility of the degradation of the LCER structure during repeated heating cycles in the presence of the abundant hydroxyl groups. As the LC monomer itself contains the ester bonds, theoretically, there is a chance that the LC domains themselves can undergo transesterification and degrade their structure in such a process. However, considering the self-ordering nature of the mesogens, this should be avoidable for two main reasons. Firstly, the obtained network has two general kinds of ester bonds: those from the LCER monomer and those created during curing from the epoxy and anhydride/acid groups. Considering the probability of the transesterification, the more probable ester to reform is the one created during the network formation, due to the geometrical and steric reasons. Those ester groups, placed solely in the elastic, purely aliphatic vicinity are more prone to contact with the free hydroxyl group, especially because every formed ester can have a residual -OH function coming from the epoxy group breakage as well as remaining hydroxyl groups from the glycerol in the close vicinity. On the other hand, the ester groups from the structure of the MU22 monomer are concealed inside the cured network and are additionally protected by the self-ordering of the mesogens into the layered domains, as has been determined in the past [46]. Both factors make them extremely less spatially available than the first kind of esters described. Secondly, the existence and stability of the LC domains is also protected differently. As the mesogenic core, and not the aliphatic spacer, is the source of the liquid crystallinity, even in the less probable event of the transesterification of the MU22 ester groups, the LC nature will remain present because of the stability of the aromatic core. The repeating heating cycles can, at most, slightly change the structure of the network due to the transesterification, but the design of the vitrimer itself is based on such changes, so it should not be considered as a problem.

Using the glycerol as a transesterification promoter can bring one more threat. It may increase the hygroscopicity of the network, which can be damaging in some applications. However, the glycerol used in the formulation does not remain as an encapsulated polyhydroxyl alcohol, but as a part of the hydroxyl-functionalized network, which may increase the hydrophilic character of the polymer, but should not promote hydrolysis and water uptake, especially when this effect is countered by the incorporation of the liquid crystalline epoxy resin MU22. MU22 contains a rigid aromatic mesogenic structure and relatively hydrophobic hydrocarbon segments. Importantly, the hydrophobic effect of MU22 is supported by previous experimental work on epoxy-based coatings, in which the incorporation of MU22 was reported to increase the hydrophobicity of the resulting coating [49]. The hydrolysis is an even smaller concern, with the research presented in [50], when the hydrolysis of the epoxy-anhydride network was possible only at high temperature and in the presence of the tertiary amine, which is not included in the polymers investigated in this paper.

### 4.4. Determination of the Optimal Glycerol Content

Surprisingly, the results show that there is a threshold of glycerol amount that allows the vitrimer to undergo complete healing (Figure 10). The best amount is the 0.5 molar ratio of the glycerol to the epoxy group amount, because the scratch disappeared at the lowest temperature (70 °C) and the healing was very fast after reaching 60 °C. The Gl-0.25 sample was also completely restructured, with slightly worse results (healing at 85 °C). The biggest amount of the glycerol (0.75 molar ratio) was unable to conduct full healing. This is probably due to the significant abundance of the free hydroxyl groups spatially blocking the contact between them and the ester groups ready for transesterification. The Gl-0.75 sample was also very difficult to handle due to its sticky, gel-like form.

The DSC results (Figure 11 and Figure 12) are interesting and they show that there is no big difference between the 0.25 and 0.5 molar ratio of glycerol and the epoxy group amount. The *T_g_* differs between them by less than 2 °C in the first and 3 °C in the second heating run. However, for the Gl-0.75 sample the *T_g_* is visibly lower in both heating scenarios, and the DSC curve presents a more complex post-curing image. This proves the different nature of the network and possibly explains the worse healing properties. Other effects on the DSC results are similar to those described before—there are some residual processes occurring during the first heating and a change in the slope of the curve in the second heating. There is a visible rise in the *T_g_* explained by the dehydration process [38]. The summarized *T_g_* values are presented in Table 3.

Worth noting is the difference between samples with a 0.5 glycerol molar ratio between batches of the vitrimers. The amount of the prepared mixtures was small and the mixing was performed by hand, so the small discrepancies are expected and should not be considered as an error, especially when a complex dehydration process occurs simultaneously.

## 5. Conclusions

The attempts to synthesize liquid crystalline epoxy vitrimers were successful, demonstrating the feasibility of incorporating LC resins into vitrimer networks. The addition of the MU22 resin resulted in a decrease in the glass transition temperature of the obtained materials, with substantial differences observed between the first and second heating cycles after synthesis, which was explained by the dehydration process. Compositions containing maleic anhydride exhibited generally better self-healing properties. The optimal glycerol content was determined as a 0.5 stoichiometric ratio relative to the total epoxy group content. Importantly, the incorporation of LC resin facilitated the healing process even in the absence of glycerol. Further optimization of curing conditions and component ratios may enable the development of materials with improved performance and enhanced stability of the polymer networks.

The approach presented in this paper offers a heavy-metal-free solution for expanding the lifespan of various equipment and machinery exposed to corrosive and damaging environments. This may bring both environmental and economic benefits.

## Figures and Tables

**Table 1:** Mass of the reagents in the investigated compositions.

Composition	EP6, g	MA, g	PA, g	MU22, g	Gl, g
EP6/MA	2.4992	1.2873	0.0000	0.0000	0.0000
EP6/MA/Gl	2.5000	1.2870	0.0000	0.0000	0.6051
EP6/MA/MU22	2.5091	1.3557	0.0000	0.2511	0.0000
EP6/MA/MU22/Gl (Gl-0.50)	2.4998	1.3555	0.0000	0.2504	0.6366
EP6/MA/MU22/Gl-0.25	2.5021	1.3549	0.0000	0.2510	0.3180
EP6/MA/MU22/Gl-0.75	2.4985	1.3552	0.0000	0.2506	0.9551
EP6/PA	2.5000	0.0000	1.9411	0.0000	0.0000
EP6/PA/Gl	2.5018	0.0000	1.9403	0.0000	0.6044
EP6/PA/MU22	2.4992	0.0000	2.0503	0.2498	0.0000
EP6/PA/MU22/Gl	2.5003	0.0000	2.0500	0.2517	0.6400

**Table 2:** Glass transition temperatures of the vitrimers.

Composition	*T_g_*, 1st Heating, °C	*T_g_*, 2nd Heating, °C
EP6/MA	38.2	104.1
EP6/MA/Gl	45.5	86.6
EP6/MA/MU22	33.3	67.8
EP6/MA/MU22/Gl	28.4	77.5
EP6/PA	108.9	107.4
EP6/PA/Gl	53.8	86.9
EP6/PA/MU22	61.4	101.4
EP6/PA/MU22/Gl	54.3	80.0

**Table 3:** *T_g_* of the MA-containing vitrimers with different glycerol content.

Composition	*T_g_*, 1st Heating, °C	*T_g_*, 2nd Heating, °C
EP6/MA/MU22/Gl-0.25	39.6	93.1
EP6/MA/MU22/Gl-0.5	38.1	90.6
EP6/MA/MU22/Gl-0.75	16.9	68.6

## Data Availability

The original contributions presented in this study are included in the article. Further inquiries can be directed to the corresponding author.

## Abbreviations

The following abbreviations are used in this manuscript:

DSC	Differential scanning calorimetry
HS-POM	Hot-stage-polarized optical microscopy
LCER	Liquid crystalline epoxy resin
Gl	Glycerol
MA	Maleic anhydride
PA	Phtalic anhydride
MU22	bis[4-(10,11-epoxyundecanoyloxy)benzoate] *p*-phenylene
*T_g_*	Glass transition temperature
